# A Scoping Review of Neuromodulation Techniques in Neurodegenerative Diseases: A Useful Tool for Clinical Practice?

**DOI:** 10.3390/medicina57030215

**Published:** 2021-02-27

**Authors:** Fabio Marson, Stefano Lasaponara, Marco Cavallo

**Affiliations:** 1 Research Institute for Neuroscience, Education and Didactics, Fondazione Patrizio Paoletti, 06081 Assisi, Italy; f.marson@fondazionepatriziopaoletti.org; 2 Department of Human Neuroscience, Sapienza University of Rome, 00185 Rome, Italy; 3 Department of Psychology, Sapienza University of Rome, 00185 Rome, Italy; stefano.lasaponara@uniroma1.it; 4 Department of Human Sciences, LUMSA University, 00193 Rome, Italy; 5 Faculty of Psychology, eCampus University, 22060 Novedrate, Italy; 6 Clinical Psychology Service, Saint George Foundation, 12030 Cavallermaggiore, Italy

**Keywords:** Alzheimer’s disease, cognitive impairment, frontotemporal dementia, neuropsychology, Parkinson’s disease, rehabilitation, stimulation

## Abstract

*Background and Objectives:* Neurodegenerative diseases that typically affect the elderly such as Alzheimer’s disease, Parkinson’s disease and frontotemporal dementia are typically characterised by significant cognitive impairment that worsens significantly over time. To date, viable pharmacological options for the cognitive symptoms in these clinical conditions are lacking. In recent years, various studies have employed neuromodulation techniques to try and contrast patients’ decay. *Materials and Methods*: We conducted an in-depth literature review of the state-of-the-art of the contribution of these techniques across these neurodegenerative diseases. *Results*: The present review reports that neuromodulation techniques targeting cognitive impairment do not allow to draw yet any definitive conclusion about their clinical efficacy although preliminary evidence is very encouraging. *Conclusions*: Further and more robust studies should evaluate the potentialities and limitations of the application of these promising therapeutic tools to neurodegenerative diseases.

## 1. Introduction

Neurodegenerative diseases targeting the elderly are a public health priority throughout the world with significant medical, psychological and economic repercussions. The most common disorders are Alzheimer’s disease (AD), frontotemporal dementia (FTD) and Parkinson’s disease (PD). Their prevalence and incidence had dramatically increased with age over the last decades, and they are expected to continue to grow due to the continuous increase in the average length of life in most countries. Neurodegenerative diseases are not homogeneous in their clinical profiles and underlying pathophysiology, although they are typically characterised by significant cognitive impairment. Time and accuracy of diagnosis are crucial factors, as they would allow the planning of timely and appropriate clinical management. As no effective pharmacological treatments for cognitive and motor symptoms are currently available, in recent years various studies had started to investigate the potential contribution of neuromodulation techniques (such as non-invasive brain stimulation techniques, NIBS) in contrasting patients’ decay. After a presentation of the most prominent epidemiological and clinical features of each disorder, the present in-depth review reports the state-of-the-art neuromodulation techniques studies targeting cognitive impairment in neurodegenerative diseases. Our twofold aim is to show the preliminary evidence currently available in the field, and to suggest that further research should evaluate the potentialities and limitations of these promising therapeutic options.

### 1.1. Clinical Profiles of Neurodegenerative Diseases

Alzheimer’s disease (AD) is a pervasive neurodegenerative disorder that represents more than 60% of dementia diagnoses among the elderly [1]. The neurophysiology of AD is mainly characterised by the extracellular accumulation of amyloid-β peptide (Aβ) plaques and intra-cellular neurofibrillary tangles containing phosphorylated tau protein on cortical and sub-cortical regions [2,3]. These local abnormalities challenge large-scale cerebral integrity, causing global white and grey matter atrophy involving the frontal regions, cingulate and temporal cortex and precuneus, selective hippocampal atrophy and increased ventricular volume [4,5,6]. Large-scale neural circuitry damages are likely to underlie clinical symptoms in AD as treatments aimed to reduce amyloid accumulation revealed to be ineffective on the reduction of cognitive and memory decline [7]. The onset of these neurophysiological manifestations precedes the onset of behavioural and psychiatric clinical symptoms, so their early detection is a crucial diagnostic factor [8]. The first noticeable cognitive changes involve progressive memory loss, impaired retrieval of semantic knowledge, reduced visuospatial attention and topographical disorientation [9,10,11], especially in early-onset AD [12]. Then, the disease progression spreads the abnormalities on a large-scale level involving long-range networks [3], causing severe impairment to executive functions [13] and anterograde amnesia [14]. A definitive cure for AD has not been found yet, since the aetiology is still unknown and the pathogenesis is unclear (for a review see [15]). For this reason, the main therapeutic protocols can only try to attenuate disease progression by reducing symptoms or delaying their onset to maintain a sufficient level of physical, psychological and social functioning [16].

Fronto-temporal dementia (FTD) is a neurodegenerative disorder which shares different commonalities with AD but, differently from AD does not involve hippocampal deterioration, thus preserving episodic and autobiographical memories [17,18]. Clinical manifestations of FTD are heterogeneous, but it is possible to identify two main variants: the behavioural variant (bvFTD) and the primary progressive aphasia (PPA) which is in turn divided in a semantic variant (svPPA), a non-fluent variant (nfvPPA) and a logopenic variant (lvPPA). The behavioural variant (bvFTD) is characterised by the deterioration of frontal and prefrontal cortices which determine behavioural abnormalities and impairments of executive functions and working memory [19] as well as attentional deficits, perseverative behaviours and mental rigidity [20,21]. In the latter stages of disease progression, the involvement of the DLPFC leads to significant deficits in planning and organization abilities [22]. The semantic variant (svPPA) is characterised by degeneration of the left anterior, middle and inferior temporal cortices [23,24] which is related to loss of word meaning [19]. Core symptoms of svPPA include loss of semantic memory in both verbal and non-verbal domains, difficulties in recognising the names and faces of known people, anomia, reading and spelling difficulties. Impairment in performing non-verbal tasks suggest that svPPA is a disease which affects the integrity of semantic knowledge rather than a purely language-related condition [25]. The non-fluent/agrammatic variant (nfvPPA) is characterised by cortical atrophy in the left inferior frontal gyrus, premotor cortex and anterior insula [26]. This atrophy causes agrammatic speech, deficits in the comprehension of syntactically complex sentences and apraxia of speech while the semantic meaning of single words are usually preserved [18,27]. The third type of PPA is called logopenic PPA (lvPPA). This form is characterised by atrophy of the left posterior temporal cortex and inferior parietal lobe, resulting in anomia, dysfluency, impaired repetition of sentences, simplified yet preserved grammar and impairments at the phonological and syntactic level of lexical processing [18].

Parkinson’s disease (PD) is classically characterised by a series of motor impairments that includes tremor, akinesia, rigidity and postural instability. It has also been extensively demonstrated that cognitive decline is a major, and often even more debilitating, symptom of PD [28]. In many cases, impairment in the cognitive domain could be typically classified as full-blown mild cognitive impairment (MCI) [29]. Neuropsychological examination of the cognitive functions in PD patients usually reveals mild to moderate deficits in the visuospatial domain, attention, working memory (WM), emotional processing [30] and general decrease in executive functions [31].

### 1.2. Neurostimulation Techniques Overview

Technological achievements have recently made available potentially useful innovative tools to researchers and clinicians. For the aim of the present review, we will now focus on neurostimulation techniques. Neuroplasticity is one of the main targets of different cognitive, physical, pharmacological and neurostimulation protocols [32]. Neuroplasticity can be induced through direct stimulation of target brain regions through different non-invasive brain stimulation techniques (NIBS). These techniques can stimulate the brain by providing magnetic stimulation (TMS) or direct current (tDCS) and alternating current (tACS) from outside the skull. Because of their power to directly modulate cerebral activity, NIBS techniques have been widely used in treatments of neurological disease involving disruption or aberration of cerebral activity [33,34,35]. As we already observed, these techniques can be adopted in conjunction with other cognitive training or with electric neurofeedback protocol to modulate specific brain regions activities [36].

Transcranial magnetic stimulation (TMS) is a technique based on the perturbation of neurophysiological activity inducing a current through a non-invasive magnetic pulse over the skull [37]. TMS can be applied adopting different approaches, such as delivering single pulse, paired pulses or multiple pulses.

Another way of stimulating brain plasticity is via the “transcranial electrical stimulation” which refers to transcranial direct current stimulation (tDCS) and transcranial alternating-current stimulation (tACS). These two techniques are based on the same principle: current flow from an electrode to another inducing electrophysiological modulation in the targeted brain region [38]. The difference between tDCS and tACS is that the former provides a stable current over time and its excitatory or inhibitory modulation of the membrane potentials’ excitability threshold depends on which electrode (anodal or cathodal, respectively) is located over the target region [39], while the latter provides a current that varies rhythmically above and below zero over time with a specific amplitude and frequency stimulating oscillatory activity. However, to date, little is known about the precise electrophysiological correlates and the specific mechanism underlying tACS effects. Two currently accepted hypotheses suggest that tACS directly entrains underlying brain oscillations and/or that tACS leads to synaptic changes via spike-timing dependent plasticity mechanisms [40].

## 2. Methods

An EBSCO-, Google Scholar- and PubMed-based literature review on neuromodulation studies targeting neurodegenerative diseases was conducted. Combinations of keywords entered for enquiries were: “transcranial magnetic stimulation” OR “transcranial direct current stimulation” OR “brain stimulation” AND ‘‘Alzheimer’s disease’’ OR ‘‘Fronto-temporal dementia’’ OR ‘‘Parkinson’s disease’’. The review was further extended by considering all of the relevant articles reported in the references of each paper. Analysis has been primarily focused on methods regarding brain stimulation, patients’ characteristics, presence/absence of cognitive symptoms, study design and experimental protocols, quantification of stimulation parameters of interest and brain imaging data, where available. We excluded research on healthy subjects only and/or conducted in non-human animals. As this field of applied clinical research is innovative and thus one cannot expect to find a number of large randomized controlled clinical trials to be definitely assessed in terms of their efficacy, we also included in our review well-conducted small-scale studies that represent the majority of studies conducted so far.

## 3. State-of-the-Art

### 3.1. Alzheimer’s Disease

Considering its incidence and prevalence, AD is one of the most studied neurodegenerative disorder. A vast literature addressing possible therapeutic options to reduce patients’ cognitive impairment is thus available.

#### 3.1.1. Transcranial Magnetic Stimulation—TMS

Single-pulse TMS (spTMS) is commonly used to probe the circuit or understand plasticity and physiological response. For clinical purposes, it has been mostly used for the early detection of AD [41], early diagnosis of dementias and MCI [42,43] and to predict the progression of cognitive decline [44,45]. However, since it is limited in its ability to elicit long-term modulation of cortical excitability, it is not commonly used for therapeutic purposes.

Repetitive TMS (rTMS) is a protocol in which trains of multiple magnetic pulses are delivered at specific frequencies and time delays to produce long-lasting perturbation of cerebral activity [46]. Theta burst stimulation is a specific type of TMS that can be applied using different (e.g., continuous or intermittent) protocols, and it is reasonably assumed to represent neural learning in a Hebbian form of long-term synaptic plasticity. The modulatory effect of this kind of cerebral stimulation depends on the coil shape, which affects the depth of the stimulated location [47] together with the intensity, duration and frequency at which pulses are delivered [48,49]. The currently most used rTMS protocol for long-lasting modulation of cerebral excitability is the high-frequency rTMS (HF-rTMS; for a review, see [50]), which consists of the delivery of one or more trains of stimuli at frequencies greater than 1 Hz. The HF-rTMS protocol showed greater efficacy and duration over time when compared to the low-frequency (LF; <1 Hz) rTMS [51]. However, the difference in effectiveness observed between these two kinds of rTMS protocols could depend on the stimulated hemisphere, since excitatory HF-rTMS and inhibitory LF-rTMS activity could be used differentially on the two hemispheres to compensate dysbalanced interhemispheric interactions.

Since most of the cognitive functions impaired in AD are related to memory recall, problem-solving, reasoning and emotional control, prefrontal regions are the main targets of NIBS. Turriziani et al. [52] stimulated the right dorsolateral prefrontal cortex (DLPFC) with LF-rTMS for 10 min before a non-verbal recognition memory task. They observed improved memory performances following the real stimulation on the right DLPFC compared to the right-sham stimulation. In contrast, no improvement has been observed in the stimulation of the left DLPFC. In a second crossover experiment, they stimulated the right DLPFC five days/week for two weeks and found that improvements persisted for at least four weeks after the end of the treatment.

A recent systematic review [53] showed the effects of HF-rTMS prolonged administration for the treatment of different neurological and psychiatric disorders, including AD. Two studies explored the clinical effect of HF-rTMS at 20 Hz over the DLPFC, stimulating only the left hemisphere for 20 sessions [54] or left and right DLPFC for 13 sessions [55]. In the first case, post-training improvement at the behavioural level has been observed on the Behavioral Pathologies in Alzheimer’s disease Rating Scale (BEHAVE-AD) as well as improvement in cognitive functions assessed with the Alzheimer’s Disease Assessment Scale-Cognitive Subscale (ADAS-Cog) [54]. In the second case, they did not observe any significant improvements at the end of the four weeks of training, but they observed improvements on Montreal Cognitive Assessment (MoCA) scores during weeks two and three [55].

Most studies have been conducted using an eight-shape coil, which can only stimulate the superficial part of the cerebral cortex, while other coils can stimulate brain regions located deeper by a factor of three [56]. This kind of deep stimulation is named deep TMS (dTMS). Avirame et al. [57] used dTMS at 10 Hz to stimulate deep prefrontal bilateral hub regions in 20 sessions in patients with moderate to severe AD. The cognitive assessment has been performed before and after the treatment using a computerised cognitive test (Minsdtreams; NeuroTrax Corp., Bellaire, TX, USA) and the Addenbrooke Cognitive Examination (ACE). Pre and post comparisons showed near-threshold improvements in both assessments, but only patients who obtained lower scores on ACE (<50) showed significant improvements at the end of the training. Moreover, changes in ACE scores were negatively correlated with baseline scores, suggesting that dTMS bilateral intervention could be particularly valuable in patients showing severe impairments.

The DLPFC is the main but not the only target region for rTMS. Precuneus is a ventral superior parietal region involved in episodic memory, visuospatial processing and global state of consciousness since it is a functional core of the default mode network (DMN; [58]). Connectivity alteration in the DMN and other networks has been observed during early-stage AD [59]. Koch et al. [60] used TMS to stimulate precuneus in 14 early-stage AD patients using 20 Hz HF-rTMS on left precuneus for a total of 20 sessions in two weeks. At the end of the training, they observed a selective improvement in episodic memory comparing real stimulation to a sham condition.

Some studies explored the effect of rTMS on different locations that are thought to underlie cognitive functions involved in AD symptomatology. Lee et al. [61] stimulated 27 probable AD patients at different brain regions for six weeks using 10 Hz-rTMS combined with a cognitive task. Locations of stimulation were divided into two clusters composed of three regions each, and their stimulation was alternated during each of the six weeks. This kind of protocol has been implemented in a dedicated system for the administration of rTMS combined with computerised cognitive training (CCT) named NeuroAD System (Neuronix Ltd., Tel Aviv, Israel). This system integrates neuronavigated TMS and CCT and is revealed to be an effective low-risk therapeutic instrument. Cognitive tasks were chosen for each session according to the cognitive function subtended by the stimulated brain regions. After the training, patients showed significant improvement in memory, language and especially in the overall ADAS-Cog score. Similar results with a similar approach have also been observed by Rabey and Dobronevsky [62].

In a recent study, the stimulation of similar regions on the different daily session has been adopted by Sabbagh et al. [63] in a large sample of AD patients including 131 participants. After 30 sessions of 10 Hz HF-rTMS with CCT, they observed improvements in ADAS-Cog and Clinical Global Impression of Change scale (CGIC) scores not immediately after training but five weeks after the end of the treatment. Their results showed that their protocol was particularly effective with mild AD patients showing baseline ADAS-Cog scores <30.

Finally, the long-term effects of rTMS have been explored by Nguyen et al. [64]. In their study, they used the aforementioned NeuroAD system combined with additional rTMS trains of pulses provided at 10 Hz during a memory task. They observed an improvement on the ADAS-Cog scale immediately after the end of the training. However, at a six-month follow-up, this improvement was maintained by only the five patients that showed greater post-training improvements. In a subsequent open-label study, they recruited the five patients that showed fewer improvements at the six-month follow-up and administered to them another two weeks of rTMS [65]. This additional intervention led to a reduction of cognitive decline and a decrease in behavioural symptoms such as apathy. This study suggests that, in some patients, five to six weeks of rTMS combined with CT could lead to cognitive improvements lasting for one year. Table 1 contains the main information about the studies reviewed.

#### 3.1.2. Transcranial Direct Current Stimulation—tDCS

The most used transcranial electrical stimulation technique in AD treatment is the tDCS in its anodal configuration. However, most of these studies are single-case or pilot studies that show encouraging but necessarily preliminary results [76,77].

Some of the first evidence for the potential therapeutic benefits of tDCS comes from a study employing anodal tDCS on bilateral temporal regions for 30 min daily for five days [78]. In this study, an improvement in visual recognition memory performance persisted for one month after the treatment had been observed. More recently, another study adopting ten sessions of anodal tDCS for 20 min on the left and right temporoparietal regions showed improvements in mini-mental state examination (MMSE), in the clock-drawing test and in the MoCA scores only in the real-tDCS group, together with an improvement in Cornell Depression Scale scores in both real-tDCS and sham-tDCS groups [79]. Finally, application of home-based anodal tDCS has been adopted by Im et al. [80]. In this study, anodal tDCS was administered for a prolonged period of six months to patients divided into a real stimulation group and a sham group. After this period, MMSE scoring and the Boston naming test performance were observed. Moreover, a marginal stabilisation of performance in some executive functions was observed compared to a general decline observed in the sham group.

However, the causal effect of tDCS has not been confirmed across all studies. For example, Cotelli et al. [81] studied the effect of tDCS on the left DLPFC for 25 min in 10 sessions. They divided the sample into three groups: one with real tdCS + CCT memory task, one with placebo tDCS + CCT memory task and a tDCS + motor training. Their results showed an improvement in face–name association test performances in both groups performing CCT memory task regardless of the tDCS protocol, showing no additive effect of real tDCS application.

Another study adopting six sessions of anodal tDCS on the left DLPFC for 20 min in two weeks failed to observe significant differences between real tDCS and sham in apathy scores, neuropsychiatric inventory (NPI), ADAS-Cog and Cornell depressive scale [82]. Authors suggested that the lack of significant results could be mainly caused by the moderate AD stage of their patients which could impair neuroplasticity mechanisms [83,84] together with the limited number of tDCS sessions adopted in the study. Similar inconclusive results have been observed by Bystad et al. [85] stimulating the left temporal lobe for 30 min over 10 sessions. Despite an observed tendency of enhanced delayed recall performance in the real tDCS group compared to placebo, in general, they failed to find significant differences in memory performances between the groups. This result may be caused by individual differences such as skull thickness, which can influence treatment effectiveness [86], and because their sample was composed of patients with AD in an advanced stage which seems to reduce positive therapeutic outcomes [87].

Despite some encouraging evidence, results of tDCS-based treatments are not always consistent across studies, highlighting the need for a larger sample size, integration with precise neurophysiological measures to better define the target of the treatments, and more coherence in experimental designs in terms of the duration and number of stimulation sessions and uniformity of clinical outcomes to obtain a clearer picture of tDCS efficacy in AD [88,89]. Table 2 contains the main information about the studies reviewed.

#### 3.1.3. Transcranial Alternating Current Stimulation—tACS

Although tACS showed potential in entraining specific frequency bands resulting in the modulation of cognitive functions in healthy subjects, only recently, a few efforts have been employed in the administration of this technique to AD patients. It is known that gamma oscillatory activity is abnormal in AD patients [106]. Gamma activity has been linked to cortico-cortical communication, multisensory processing and integration across different brain regions [66,107]. For this reason, Naro et al. [108] stimulated six different regions in the left hemisphere using tACS to evaluate gamma frequency entraining in AD, MCI and healthy participants. Results showed that AD patients did not show any tACS modulatory effect compared to MCI and healthy participants. Interestingly, MCI patients with AD-similar gamma profiles developed AD within two years from the end of the training, suggesting that gamma tACS could be used as a potential early diagnostic tool for AD.

Some optogenetic studies suggested that externally driven gamma activity could reduce Aβ depositions and p-Tau levels [109]. In a recent study, Dhaynaut et al. [90] used tACS in the gamma frequency range (40 Hz) for 20 sessions (one hsession) on bilateral temporal lobes. After the tACS treatment, a trend of a decrease of intracerebral p-Tau has been observed, especially on temporal lobes, suggesting a potential novel therapeutic approach for neurophysiological AD manifestation.

#### 3.1.4. Other Neurostimulation Techniques

Besides TMS as mentioned above and tDCS and tACS, there are other less diffused novel NIBS techniques that can directly or indirectly modulate cerebral activity such as the radio-electric asymmetry conveyer (REAC), focused ultrasound (FU) and transcranial pulse stimulation (TPS) with ultrasounds. The radio-electric asymmetry conveyer (REAC) is a biomedical device that allows the induction of a small current in a portion of biological tissue through the emission of very weak microwaves in the Wi-Fi frequency range [110]. These microwaves can induce small changes in cerebral activity that can last for a prolonged time after stimulation. These new non-invasive neurostimulation techniques have also been used in AD patients through stimulation of the ear lobe with a series of 500 ms radio-frequency bursts. Patients underwent two cycles of treatment consisting of 18 sessions each cycle, with an average time delay of six months between cycles. After the first cycle, there was an improvement in all the cognitive and behavioural functioning indices (i.e., MMSE; NPI; activity of daily living, ADL; and instrumental activity of daily living, IADL). Further improvements in all these indices, except ADL, have been observed after the second cycle of treatment [111].

One of the most limiting factors of pharmacological treatments is that most of the chemical particles in the blood flow are not able to pass the brain–blood barrier, a regulatory interface that determines the entrance of substances in the brain [91]. Focused ultrasound (FU) is a non-invasive stimulation that can selectively, transiently and safely force the opening of the blood–brain barrier to increase the blood flow in specific brain regions and allow the passage of drugs. Recently, some studies successfully adopted FU to open the brain–blood barrier in human AD patients [112,113,114]. Two of these studies demonstrated that the application of FU on the white matter in the right prefrontal cortex could safely cause an opening of the brain–blood barrier lasting for 24 h [113] and a reduced resting-state functional connectivity in the ipsilateral frontoparietal network lasting for the same time [112]. The other study reported a selective opening of the brain–blood barrier in the hippocampal and entorhinal cortex, demonstrating that it is possible to modulate the brain–blood barrier permeability in the very specific structure of the human brain to deliver pharmacological treatments directly to target regions without observing significant clinical worsening and aversive side-effects. However, the adoption of such techniques and its potential therapeutic implication need much more studies to provide precise and reliable therapeutic protocols. Finally, among the ultrasound-based brain stimulation techniques, there is also a clinical sonication technique based on single ultrashort ultrasound pulses (transcranial pulse stimulation, TPS). This was recently used in a study by Beisteiner et al. [115] in which ultrasound brain stimulation and first observations of long-term effects are presented. In this study, the authors included simulation data, laboratory measurements with rat and human skulls and brains, and finally, in vivo modulations of somatosensory-evoked potentials in healthy subjects (sham-controlled) and 35 patients with Alzheimer’s disease acquired in a multicenter setting. The results showed large safety margins and dose-dependent neuromodulation. A high treatment tolerability and no major side effects were reported. Neuropsychological scores improve significantly after TPS treatment and improvement lasts up to three months and correlates with an upregulation of the memory network, as revealed by fMRI data. These results encourage broad neuroscientific application and translation of the method to clinical therapy and randomized sham-controlled clinical studies. Table 3 contains the main information about the studies reviewed.

### 3.2. Frontotemporal Dementia

NIBS techniques have only recently been applied to PPA treatments. The increasing evidence about the efficacy of neurostimulation techniques in treating neurodegenerative disorders can be inferred by the growing number of studies, reviews and meta-analyses published in recent years [107,116]. However, most of the studies in the literature adopted rTMS or anodal tDCS with or without language training. To the best of our knowledge, no studies so far have explored the use of tACS in PPA.

#### 3.2.1. Transcranial Magnetic Stimulation—TMS

One of the first studies on the effect of TMS with PPA has been performed by Finocchiaro et al. [66]. This study explored the effect of HF-rTMS over the left anterior midfrontal gyrus on a patient affected by a non-specified PPA. The patient performed different assessments with two sentence-completion tasks with missing verbs, two sentence-completion tasks with missing nouns and a memory span task at baseline, after a period of rTMS stimulation, after sham stimulation and after a final period of rTMS stimulation again. During real or sham stimulation, the patient did not receive any linguistic training. Results showed that the patient improvement lasted for 60 days after the first rTMS session and 45 days after the second rTMS session, while after SHAM, his performances were not different from baseline. Authors attributed the observed benefits to increased excitability of the left prefrontal cortex, whose functions are directly or indirectly involved in language processing, as also observed by Beeson et al. [117].

An approach similar to Finocchiaro et al. has been more recently adopted by Bereau et al. [67]. A patient with lvPPA received HF-rTMS at 10 Hz over the left DLPFC for one week with two sessions per day. Neuropsychological measurement of cognitive functions, verbal comprehension, a picture-naming test, verbal repetition and other phonological and categorical fluency tests and indexes of cerebral perfusion using a single-photon emission computerised tomography (SPECT) scan were performed before and after the treatment. The patient showed improved processing speed and language skills such as non-word repetition, phonological and categorical fluency. Improvements in verbal fluency and reduced paraphasia were observed three months after the end of the training. Together with these benefits, they also observed an increase in the left fronto-temporoparietal and striatum perfusion one month after the end of the treatment.

Another study [68] employed an H-shaped coil to deeper stimulate the left DLPFC using HF-rTMS at 20 Hz. A patient diagnosed with lvPPA received a total of two real and two sham stimulation sessions. Each session involved 20 min of stimulation per day for five consecutive days with an inter-session interval of 14 days. A neuropsychological battery including tests of cognitive functioning, verbal fluency and a creative writing task were administered before, immediately after and seven days after each TMS session. Whereas cognitive tests showed no changes following any TMS session, language-related tests showed significant improvement in verbal fluency and a decreased number of errors in written texts following real stimulation but not after sham stimulation. However, these benefits seemed to disappear within seven days.

These single case studies explored the effect of rTMS over left prefrontal regions showing language-specific improvements in PPA patients despite stimulation not being accompanied by specific language treatments. However, a few recent studies have combined TMS with language-related training. The administration of rTMS over the left and right DLPFC in 10 nfvPPA patients seemed to facilitate online performance in an action naming task [69]. In another pilot study, Margolis et al. [70] adopted HF-rTMS at 20 Hz to stimulate the right and left DLPFC during an online action/object naming task performed by eight patients diagnosed with nfvPPA. Moreover, global cognition and fluency were assessed at baseline and after each rTMS session using the Montreal Cognitive Assessment (MOCA) and the letter fluency task, respectively. They observed improvements in the action naming task, replicating the results of the previous study [69]. Moreover, they observed an increase in MoCA scoring following experimental sessions and an almost significant improvement in the letter fluency task. Interestingly, these effects were associated with the stimulation of the left DLPFC, while the stimulation of the right DLPFC was associated only with improved post-stimulation MoCA scores.

Stimulation of bilateral DLPFC in patients with different subtypes of FTD has been adopted in another recent open-label pilot study [71]. After ten daily sessions of 10 Hz HF-rTMS, patients showed improvements in letter and digit cancellation, speed of reading, Stroop test and MoCA scores. A large portion of these patients were diagnosed with bvFTD. Their improvements were comparable with other FTD subtypes, suggesting that stimulation of DLPFC could be valuable also in treating cognitive and linguistic symptoms in the behavioural variant. To the best of our knowledge, this is the only study in which bvFTD patients were involved.

In general, rTMS studies yielded promising results about the online enhancement of linguistic and cognitive abilities in PPA and a few bvFTD patients, even if their offline duration after stimulation is still a matter of debate and further studies are required. Stimulation of the DLPFC seems to support linguistic abilities and lexical retrieval, especially in patients whose semantic knowledge is not degraded, such as in nfvPPA patients. Table 1 contains the main information about the studies reviewed.

#### 3.2.2. Transcranial Direct Current Stimulation—tDCS

tDCS has been classically applied in a post-stroke aphasic patient [118], and more recently, it has also been adopted in the treatment of PPA patients [100]. Differently from the TMS studies described earlier, different studies adopted tDCS alone or in combination with linguistic training.

Cotelli et al. [91] applied daily tDCS stimulation for 25 min on the left DLPFC for two weeks with 16 agrammatic PPA patients; eight received a real stimulation and eight received a placebo stimulation. Regardless of the tDCS protocol, all patients underwent an individualised computerised anomia training (ICAT). Improvements in naming accuracy were observed on trained and, to a lesser extent, untrained items at 12 weeks after training in the real tDCS group. Anodal tDCS is thought to improve neuronal excitability, stimulating cortical plasticity. As already observed with TMS, stimulation of the left DLPFC is associated with improvement in lexical retrieval in PPA.

Also, the inferior frontal gyrus (IFG) has been selected as a target for tDCS protocols. Tsapkini et al. [92] applied anodal tDCS on left IFG to six patients (two nfvPPA and four lvPPA), adopting a sham-controlled within-group cross-over design. Patients received 15 treatment sessions (three to five per week) of real or sham stimulation while training on a spelling task based on the grapheme-to-morpheme conversion. Clinical assessment was performed before and immediately after the training, while follow-ups occurred at two weeks and two months after the training. Improvements in spelling tasks were observed on treated items in both real and sham conditions. However, the combination of tDCS with the grapheme-to-morpheme conversion training showed a more extended duration of positive effects and a generalisation to untrained items.

Since one of the most significant limitations of the observed studies is the small sample size, the same group recently performed a study adopting the same experimental design involving a total of 36 PPA patients diagnosed with lvPPA, nfvPPA and svPPA [93]. Benefits on the production of both treated and untreated items associated with real tDCS stimulation were found, and improvements lasted for two months after treatment. Interestingly, they observed improvements in lvPPA and nfvPPA but not in svPPA.

In another cross-over sham-controlled study involving seven PPA (five nfvPPA, two svPPA) and three AD patients showing linguistic impairments, the inferior temporoparietal regions were targeted by tDCS [94]. In this study, anodal tDCS was applied in combination with a picture-naming task for ten sessions in 18 days. Results were in line with other studies showing more significant and durable improvements in a picture-naming task on trained, and to a lesser extent, untrained items when real tDCS was applied with linguistic training. At the same time, a decrease in picture-naming performance was observed in the sham condition.

Most of the studies here described adopted specific linguistic training designed to affect single features of language processing such as spelling or noun retrieval. Gervits et al. [95] adopted a different, more general approach by asking six patients with PPA (two nfvPPA and four lvPPA) to narrate a story depicted in a wordless children’s book while receiving two weeks of daily tDCS stimulation on the left frontotemporal region. Assessments were performed using a battery of linguistic tests including picture naming, speech fluency, grammatical comprehension, semantic processing and sentence repetition immediately, at six and 12 weeks after stimulation. They observed improvements in language functions such as grammatical comprehension, elicited speech rate and utterances length. Improvements were maintained up to three months after treatment. Despite the interesting training protocol and results, one of the main limitations of this study is the lack of a control group or condition.

Interesting results have been observed in a protocol adopting tDCS alone without linguistic or cognitive training. Teichmann et al. [96] performed a sham-controlled cross-over double-blind study involving 12 svPPA patients and a control group of 15 healthy participants. Stimulation consisted of 20 min of tDCS of anodal excitatory on the left temporal pole (TP), cathodal inhibitory on the right TP and sham stimulation over the left TP in different sessions. For the assessments, living and non-living items were used in either verbal or visual form. A probe item was presented on the top of the screen while a related item and a distractor were presented below. Participants were asked to select the item related to the probe. At baseline, patients showed general semantic impairments compared to controls, especially with verbal stimuli and with the living category. In the post-treatment assessment, a general improvement in performance in the verbal modality was found after both anodal-left and cathodal-right tDCS but not in sham. Interestingly, right inhibitory tDCS was associated with better performances with combined living category and verbal form and were further associated with improvements in reaction times with verbal stimuli. Improvements in verbal but not visual items contrast the hypothesis of a bilateral amodal semantic network but instead support the existence of a verbal semantic system in the left anterior temporal cortex affected by PPA [119].

Another very recent large sampled double-blind study recently explored the effect of anodal tDCS over the left prefrontal cortex without the contextual administration of linguistic training on clinical measures and intracortical connectivity measures such as intracortical facilitation (ICF) and short-interval intracortical inhibition (SICI) [97]. These intracortical connectivity measures reflect glutamatergic (ICF) and GABAergic (SICI) neurotransmission, which seems to be involved in the neurophysiological profile of FTD. A total of 70 patients diagnosed with bvFTD or PPA (55 symptomatic and 15 pre-symptomatic) underwent real tDCS stimulation or sham stimulation five days a week for two weeks. Clinical scores and intracortical connectivity measures were assessed before and after the treatment and at two follow up points at one and six months from the end of the treatment. As for clinical measures, cognitive tests such as the MMSE, Stroop test, phonemic verbal fluency test, digit-symbol substitution test, an emotion recognition test and the Cambridge behavior inventory (CBI) were administered. Both symptomatic and pre-symptomatic patients showed tDCS-related changes in intracortical connectivity measures, which have been associated with increased cortical plasticity. Together with neurophysiological changes, a trend of improvements or significant improvements in clinical scores were found both within participants (comparing post-treatments with baseline) and between participants (comparing real tDCS with sham).

Regarding bvFTD, a recent randomized, double-blind, placebo-controlled study [98] tested the hypothesis that tDCS over the medial frontal cortex (MFC) could selectively enhance communicative intention processing, which is a specific theory-of-mind (ToM) ability. The authors administered a single-session online design, in which a ToM task measuring the ability to represent other people’s private and communicative intentions was used during active or sham tDCS to 16 bvFTD patients and healthy controls. The authors observed significant and selective accuracy improvements in the comprehension of communicative intentions after active stimulation. This first study analyzing ToM ability in patients with bvFTD using tDCS stimulation could potentially contribute to the development of an effective, noninvasive brain stimulation treatment of ToM impairments in patients with bvFTD. Table 2 contains the main information about the studies reviewed.

### 3.3. Parkinson’s Disease

Since PD and its cognitive correlates have a significant impact on the health care costs as well as on the quality of life (QoL) of both patients and their caregivers, it is urgent to identify intervention strategies to slow down cognitive deterioration. To this end, pharmacological treatments have failed at specifically addressing and ameliorating cognitive symptoms in patients with PD [120], while a series of non-pharmacological approaches, consisting in cognitive stimulation or non-invasive brain stimulation, had attracted increasing interest over the last few years.

#### 3.3.1. Transcranial Magnetic Stimulation—TMS

Early investigations were focused on the possibility of ameliorating PD symptoms using non-invasive brain stimulation. These results were initially summarised in a first meta-analysis by Elahi and co-workers [121] in which the authors evaluated the effects of repetitive transcranial magnetic stimulation (rTMS) in 275 patients with PD from 10 studies. The outcome of interest was the motor section of the Unified Parkinson’s Disease Rating Scale (UPDRS) on which the authors calculated the effect size for all studies included in the meta-analysis. A general effect size of 20.58 was found in UPDRS for high-frequency rTMS studies, with no significant effects for low-frequency rTMS studies. Given these results, the authors concluded that the meta-analysis confirmed the benefit of high-frequency rTMS on motor signs in PD while lower-frequency rTMS had little effect. However, despite the presence of these early encouraging pieces of evidence suggesting the significant effectiveness of TMS and rTMS in the treatment of motor symptoms in PD, some recent investigations showed that magnetic stimulation of the motor and prefrontal cortices appears safe and improves mood, but failed to improve motor performance and functional status in PD.

In particular, Benninger et al. [72] in a randomised, double-blind, sham-controlled study, investigated the safety and efficacy of intermittent theta-burst stimulation (iTBS) in 26 patients with mild to moderate PD. Stimulation was provided over the motor and dorsolateral prefrontal cortices in eight sessions over two weeks. Assessment of safety and clinical efficacy over one month included timed tests of gait and bradykinesia, UPDRS, and additional clinical, neuropsychological, and neurophysiologic measures. The authors reported the beneficial effects of iTBS on mood, but no improvement of gait, bradykinesia, UPDRS, and other measures. EEG/EMG monitoring recorded no pathologic increase of cortical excitability or epileptic activity. Some reported discomfort or pain and one experienced tinnitus during real stimulation.

In contrast, Brys et al., [73] found that, in patients with PD and concomitant depression, M1 rTMS at a frequency of 10 Hz is an effective treatment for motor symptoms, while mood benefit after two weeks of DLPFC rTMS is not better than the sham and targeting both M1 and DLPFC in each rTMS session showed no evidence of synergistic effects.

More recently, repetitive deep transcranial magnetic stimulation (rDTMS) was used in patients with PD using the H5 coil for the low-frequency stimulation of the primary motor cortex, followed by the high-frequency rDTMS of the prefrontal cortex [74]. The main outcome measures were the total and motor scores of the UPDRS. Secondary measures included a rating of depression and quantitative motor tasks. Results revealed a significant main effect for a time between baseline and day 90 (end of treatment), indicating that there was an improvement of both scores over time in the whole sample. Indeed, simple effects analysis was significant both in the rDTMS group and reached a P-value of 0.06 in the sham group. Taken together, these findings point out that, although rDTMS treatment exhibited some motor improvements, it was impossible to demonstrate a clear advantage for real treatment over sham.

Finally, Fricke and co-workers [75] hypothesised that PD symptoms could be ameliorated by a lasting decoupling of subthalamic nucleus neurons by associative dual-site rTMS (1 Hz) employed to the primary motor cortex and dorsal premotor cortex. To this aim, 20 PD patients were treated in a blinded, placebo-controlled cross-over design. The authors reported no significant improvement in clinical outcome parameters. Furthermore, a variation of the premotor stimulation site did not induce beneficial effects either. On these grounds, the authors concluded that a successful treatment using TMS, which targets subcortical nuclei, might require intervention over several days or more detailed physiological information about the individual brain state and stimulation-induced subcortical effects. Table 1 contains the main information about the studies reviewed.

#### 3.3.2. Transcranial Direct Current Stimulation—tDCS

Various studies investigated the effects of tDCS on cognition in PD patients. It was generally observed that anodal stimulation of the dorsolateral prefrontal cortex (DLPFC) resulted in significant improvements in WM [99], phonemic verbal fluency [100] and executive functions [101]. In this latter study, changes in executive functions were measured by the Trail Making Test, and it was also showed that benefits deriving from tDCS stimulation lasted after the one-month follow-up. More recently, in agreement with these results in a double-blind, randomised and sham-controlled study, a 20 min at two mA stimulation of the DLPFC was given to twenty participants who were tested before and after stimulation with the Trail Making Test (TMT), verbal fluency test, Stroop test, timed up-and-go test and video gait analysis. Improvements due to stimulations were observed for the verbal fluency test and in the Stroop test [102].

A promising approach considers the possibility to integrate cognitive training and brain stimulation. To this aim, in a recent study by Lawrence et al., the authors examined the different effects on cognitive function and functional outcomes in PD patients with MCI, of standard cognitive training (1), tailored cognitive training (2), tDCS stimulation (3), standard cognitive training in association with tDCS (4), or tailored cognitive training in association with tDCS (5). In all cases, tDCS consisted of anodal stimulation of the left DLPFC. All interventions lasted four weeks, with cognitive and functional outcomes measured at baseline, post-intervention, and follow-up. Results showed that, when compared to the control group, all of the five intervention groups demonstrated variable statistically significant improvement across executive function, attention/working memory, memory, language, activities of daily living (ADL), and QoL. Most importantly, it was shown that combining tDCS with tailored/standard cognitive training provided greater therapeutic effects [103]. Similarly, in a study by Manenti et al., 22 patients with PD underwent a two-week treatment involving the daily application of active tDCS plus computerised cognitive training (CCT) or sham tDCS plus CCT. Each patient was evaluated at baseline, after treatment and at the three-month follow-up. The results pointed out that, while an improvement in general cognitive performance was observed in both groups at post-treatment and follow-up, greater and significant changes from the baseline of phonemic verbal fluency were exclusively present in the active tDCS group [104].

Finally, another study by the same group of researchers [105] investigated the effects of anodal transcranial direct current stimulation applied over the DLPFC, combined this time with physical therapy in 20 PD patients. These were assigned to one of two study groups—group 1, anodal tDCS plus physical therapy (*n* = 10); or group 2, placebo tDCS plus physical therapy (*n* = 10). The treatment, lasting two weeks, consisted of daily direct current stimulation application for 25 min during physical therapy. The long-term effects of the treatment were evaluated on clinical, neuropsychological, and motor task performance at the three-month follow-up. The authors pointed out an improvement in motor abilities and a reduction of depressive symptoms in both groups after the end of treatment and at the three-month follow-up. However, the Parkinson’s Disease Cognitive Rating Scale and verbal fluency test performances increased only in the anodal direct current stimulation group with a stable effect at follow-up.

Taken together, all of these studies showed that tDCS could produce a series of significant improvements in motor and non-motor symptoms in PD and that this may be a relevant tool to improve cognitive abilities in PD, providing a novel therapeutic strategy for patients with mild cognitive impairment. Table 2 contains the main information about the studies reviewed.

## 4. Conclusions

Neurodegenerative diseases are heterogeneous in their clinical profiles and underlying pathophysiology. In most cases, they share the presence of significant cognitive impairment, depending on the diseases themselves and their clinical stage. Due to the absence of effective pharmacological treatments for their most prominent cognitive symptoms, researchers and clinicians are in urgent need of valid tools to contrast patients’ decay. Non-invasive brain stimulation techniques such as TMS and tDCS have been shown to be safe and effective methods for improving cognitive and affective functions in neuropsychiatric disorders such as depression, anxiety and PTSD symptoms. As reviewed in the present paper, neuromodulation techniques may represent a promising tool for treating the cognitive symptoms in neurodegenerative conditions in the elderly, as the preliminary evidence provided by the pilot studies published so far is encouraging. However, as current research in the field has not reached a mature level already and thus its results should be considered necessarily as preliminary, our review points out the need for further and more robust studies including larger samples of patients and a more efficient integration of neuromodulation techniques and cognitive tools. A better definition of treatments’ targets and more coherence in experimental design and clinical outcomes will generate a clearer picture of neuromodulation techniques’ efficacy in these neurodegenerative conditions.

To conclude, at this point in time, given the absence of large and robust studies able to provide strong evidence in favor of the use of these techniques with these clinical targets, one cannot draw any definitive conclusion about their efficacy although preliminary evidence is encouraging (please refer to Table 1, Table 2 and Table 3, which show a significant improvement of patients in 34 out of the 46 studies considered). By referring to a widely accepted classifications of efficacy (e.g., grade practice recommendations), at this point in time, the level of recommendation considers these techniques a viable therapeutic option, meaning that the qualifying evidence can be classified as levels II, III or IV with findings not always consistent across all studies. However, in our view, larger and more robust studies would help to overcome some of the limitations that small-scale studies currently present. In doing so, a more evidence-based clinical reasoning will permit serious consideration of the possible integration of innovative neuromodulation techniques with more traditional interventions targeting neurodegenerative patients with cognitive rehabilitative purposes in mind.

## Figures and Tables

**Table 1:** Main information of the reviewed TMS studies.

Reference, Authors, Published Year	n	Diagnosis	Mean Age (Years) (SD)	Protocol (Name) (Parameters)	Duration (Days × Weeks)	Target Region	Study Type	Control	Cognitive Training Used	Main Results	Duration Post-Treatment
[52] Turriziani et al., 2019 (exp1)	24	AD	72.4 5.2	LF-rTMS 1 Hz	Four sessions	Left and right DLPFC	Single-blind	Crossover Sham	Non-verbal recognition memory task	Improved memory	Not tested
[52] Turriziani et al., 2019 (exp2)	14	AD	71.28 3.5	LF-rTMS 1 Hz	5 d × 2 w	Right DLPFC	Single-blind	Crossover Sham	None	Improved episodic memory	Four weeks
[54] Yue et al., 2015	54	AD	71.4 4.9	HF-rTMS 20 Hz	5 d × 4 w	Left DLPFC	Double-blind	Sham Group	None	Improved BEHAVE-AD and adas-cog	Not tested
[55] Rutherford et al., 2015	10	AD	57~87 \	HF-rTMS 20 Hz	5 d × 4 w + 5 d × 2 w	Left and right DLPFC	Double-blind + Open label	Crossover Sham	None	Improved MoCA (during the training)	Not tested
[57] Avirame et al., 2007	11	AD	76 7	Deep HF-rTMS 10 Hz	20 sessions	Bilateral DLPFC	Open-label	None	None	Improved ACE in severe patients	Not tested
[60] Koch et al., 2018	14	AD	70 5.1	HF-rTMS 20 Hz	5 d × 2 w	Precuneus	Double-blind	Crossover Sham	None	Improved episodic memory	Not tested
[61] Lee et al., 2016	26	AD	71.6 6.8	HF-rTMS 10 Hz	5 d × 6 w	Multiple sites	Double-blind	Sham Group	Multiple CCT	Improved ADAS-Cog	Six weeks
[63] Sabbagh et al., 2019	131	AD	~76 \	HF-rTMS 10 Hz	5 d × 6 w	Multiple sites	Double-blind	Sham Group	Multiple CCT	Improved ADAS-Cog and CGIC (only at follow-up)	Five weeks
[64] Nguyen et al., 2017	10	Probable AD	73 7.2	HF-rTMS 10 Hz	5 d × 5 w	Multiple sites	Open-label	None	Multiple CCT	Improved ADAS-Cog	Six months (only five patients)
[66] Finocchiaro et al., 2006	1	PPA	60	HF-rTMS	5 d × 1 w × 2	Left-Anterior MFG	Single case	Sham Condition	None	Improved verb task	Three months
[67] Bereau et al., 2016	1	lvPPA	66	HF-rTMS	10 sessions × 1 w	Left DLPFC	Single case	None	None	Improved speed processing and linguistic skills	Three months
[68] Trebbastoni et al., 2013	1	lvPPA	50	Deep HF-rTMS 50 Hz	5 d × ~14 w	Left DLPFC	Single case online	Sham Condition	None	Improved verbal fluency	Seven days
[69] Cotelli et al., 2012	10	nfvPPA	69.1 9.3	HF-rTMS 20 Hz	Single session	Left and Right DLPFC	Single-blind	Sham Condition	None	Online improvement of action naming	Not tested
[70] Margolis et al., 2019	6	nfvPPA	67 7	HF-rTMS 20 Hz	Single session	Left and right DLPFC	Single-blind online	Sham Condition	None	Online improvement of action naming	Not tested
[71] Antczak et al., 2018	11	Various FTD	61.7 10.1	HF-rTMS 10 Hz	5 d × 2 w	Left and right DLPFC	Open label	None	None	Improved MoCA, stroop and other	Not tested
[72] Benninger et al., 2011	26	PD	40–80 /	iTBS 50 Hz	4 d × 2 w	Left and right DLPFC and M1	Double blind	Sham Group	None	Slightly improved mood only	No
[73] Brys et al., 2016	61	PD + depression	55~70 /	HF-rTMS 10 Hz	5 d × 2 w	Left and right M1, DLPFC and both	Double blind	Sham Group	None	Improved motor functions only	One month
[74] Cohen et al., 2018	48	PD	65.6 7.5	Deep LF- and HF-rTMS 1 Hz & 10 Hz	~2 s × 12 w	M1 and PFC	Double blind	Sham Group	None	Slightly improved motor functions	Not tested
[75] Fricke et al., 2019	20	PD	58.5 14.1	LF-rTMS 1 Hz	2 sessions	Pre-Motor Cortex and M1	Single blind	Crossover Sham	None	No significant improvements	Not tested

AD = Alzheimer’s disease; ADAS-Cog = Alzheimer’s Disease Assessment Scale-Cognitive Subscale; CCT = computerised cognitive training; DLPFC = dorsolateral prefrontal cortex; FTD = frontotemporal dementia; lvPPA = logopenic variant PPA M1 = primary motor cortex; MFG = medial frontal gyrus; MoCA = Montreal Cognitive Assessment; nfvPPA = non-fluent variant PPA; PD = Parkinson’s disease; PPA = primary progressive aphasia.

**Table 2:** Main information of the reviewed tDCS studies.

Reference, Authors, Published Year	n	Diagnosis	Mean Age (Years) (SD)	Protocol (Name) (Parameters)	Duration (Days × Weeks)	Target Region	Study Type	Control	Cognitive Training Used	Main Results	Duration Post-Treatment
[78] Boggio et al., 2012	15	AD	~75~85 /	tDCS 2 mA 30 min	5 d × 1 w	Temporal lobes	Double-blind	Crossover Sham	None	Improved visual recognition memory	Four weeks
[79] Khedr et al., 2019	56	AD	~64.2~65.2 /	tDCS 2 mA 20 min	5 d × 2 w	Temporal lobes	Double-blind	Sham Group	None	Improved MMSE, MoCA, clock drawing	Not tested
[80] Im et al., 2019	18	AD	~71~74 /	tDCS 2 mA 30 min	7 d × 18 w	DLPFC	Double-blind	Sham Group	None	Improved MMSE and BNT	Not tested
[81] Cotelli et al., 2014	36	AD	70~80 /	tDCS 2 mA 25 min	5 d × 2 w	Left DLPFC	Double-blind	Sham Group	Memory task or Motor training	No tDCS related effect	No
[82] Suemoto et al., 2014	40	AD	80.5 7.5	tDCS 2 mA 20 min	3 d × 2 w	Left DLPFC	Double-blind	Sham Group	None	No effect	No
[77] Bystad et al., 2016	25	AD	59–83	tDCS 2 mA 30 min	Six sessions	Left Temporal Cortex	Double-blind	Sham Group	None	No effect	Not tested
[90] Dhaynaut et al., 2020	5	AD	> 65	tACS 40 Hz 1 h	20 session	Left and right Temporal Lobes	Pilot study	None	None	Trend for decrease of p-Tau	Not tested
[91] Cotelli et al., 2014	16	Agrammatic PPA	66.9 8.2	tDCS 2 mA 25 min	5 d × 2 w	Left DLPFC	Double-blind	Sham Group	ICAT	Improved naming accuracy	Three months
[92] Tsakpini et al., 2014	6	nfvPPA and lvPPA	Not reported	tDCS 2 mA 20 min	3/5 d × 3 w	Left IFG	Double-blind	Crossover Sham	Phoneme-to-grapheme task	Improved spelling on untrained items	Two months
[93] Tsakpini et al., 2018	36	Various PPA	Not reported	tDCS 2 mA 20 min	5 d × 3 w	Left IFG	Double-blind	Crossover Sham	Spelling therapy	Improved linguistic production for trained and untrained items	Two months
[94] Roncero et al., 2017	10	7 PPA, 3 AD	67.4 6.2	tDCS 2 mA 30 min	20 sessions	ITP region	Double-blind	Crossover Sham	Picture-naming	Improved picture-naming	Two weeks
[95] Gervits et al., 2016	6	nfvPPA, lvPPA	66.2 5.7	tDCS 1.5 mA 20 min	5 d × 2 w	Left fronto-temporal	Pilot Open label	None	Narration of wordless books	Improved grammar, speech rate and length	Three months
[96] Teichmann et al., 2016	12	svPPA	66.8 2.1	tDCS 1.6 mA 20 min	Three sessions	Temporal Poles	Double-blind	Crossover Sham	None	Improved verbal accuracy and speed	Not tested
[97] Benussi et al., 2020	70	bvFTD and PPA	62 7.2	tDCS 2 mA 20 min	5 d × 2 w	Left PFC	Double-blind	Sham Group	None	Improved intracortical connectivity	Six months
[98] Cotelli et al., 2018	16	bvFTD	64.9 8.6	tDCS 1.5 mA 10 min	Two sessions	MFC	Double-blind	Crossover Sham	ToM task	Improved comprehension and communication	Not tested
[99] Boggio et al., 2006	18	PD	61.1 ~10	tDCS 2 mA 20 min	Three sessions	Left DLPFC and M1	Double-blind	Crossover Sham	3-back WM task	Improved accuracy	Not tested
[100] Pereira et al., 2013	16	PD	61.5 9.9	tDCS 2 mA 20 min	Two sessions	Left DLPFC and TPC	Single-blind	Crossover No sham	None	Increased fluency and functional connectivity	Not tested
[101] Doruk et al., 2014	18	PD	61 8	tDCS 2 mA 20 min	5 d × 2 w	Left and right DLPFC	Double-blind	Sham Group	None	Improved Trail Making Test B	One month
[102] Bueno et al., 2019	20	PD	64.4 8.9	tDCS 2 mA 20 min	Two sessions	Left DLPFC	Double-blind	Crossover Sham	None	Improved Verbal fluency	Not tested
[103] Lawrence et al., 2018	42	PD-MCI	65–75 /	tDCS 1.5 mA 20 min	1 d × 4 w	Left DLPFC	Open label	Passive Group	Different CCTs	Improved cognition, ADL and QoL	Three months
[104] Manenti et al., 2018	22	PD	~63–65 ~10	tDCS 2 mA 25 min	5 d × 2 w	Left DLPFC	Double-blind	Sham Group	Different CCTs	Improved verbal fluency and reduction of depression	Three months
[105] Manenti et al., 2016	20	PD	69 8	tDCS 2 mA 25 min	5 d × 2 w	Left or right DLPFC	Double-blind	Sham Group	Physical therapy	Improved PDCRS and verbal fluency	Three months

AD = Alzheimer’s disease; ADL = Activities of daily living; BNT =Boston naming test; bvFTD = behavioural variant of frontotemporal dementia; CCT = computerised cognitive training; DLPFC = dorsolateral prefrontal cortex; FTD = frontotemporal dementia; ICAT = individualised computerised anomia training; IFG = inferior frontal gyrus; ITP = inferior temporo-parietal region; lvPPA = logopenic variant PPA; M1 = primary motor cortex; MMSE = Mini Mental State Examination; MoCA = Montreal Cognitive Assessment; nfvPPA = non-fluent variant PPA; PD = Parkinson’s disease; PD-MCI = PD-Mild Cognitive Impairment; PPA = primary progressive aphasia; QoL = quality of life; ToM = Theory of Mind.

**Table 3:** Main information of the reviewed studies applying neuromodulation techniques other than TMS and tDCS.

Reference, Authors, Published Year	n	Diagnosis	Mean Age (Years) (SD)	Protocol (Name) (Parameters)	Duration (Days × Weeks)	Target Region	Study Type	Control	Cognitive Training used	Main Results	Duration Post-treatment
[111] Mannu et al., 2011	8	AD	65.4 3.5	REAC	18 sessions × 2 cycles	\	Open label	None	None	Improved MMSE, NPI, ADL, IADL	Not tested
[113] Lipsman et al., 2018	5	AD	66.2 6.6	MRI guided-Focused Ultrasound	Two sessions	Right frontal lobe	Open label pilot	None	None	Safe opening of BBB	24hafter each session
[112] Meng et al., 2019	5	AD	66.8 6.1	MRI guided-Focused Ultrasound	Two sessions	Right frontal lobe	Open label	No treatment	None	Reduction of frontoparietal connectivity	24hafter each session
[114] Rezai et a., 2020	6	Early AD	55~73	MRI guided-Focused Ultrasound	17 sessions	Hyppocampus	Open label	None	None	Opening of hippocampal BBB	24hafter each session
[115] Beisteiner et al., 2020	35	AD	Not reported	TPS	3 d × 2–4 w	Different regions	Open label	None	None	Improved neuropsychological measurements	Three months

ADL = Activities of daily living; AD = Alzheimer’s disease; CCT = computerised cognitive training; BBB = brain–blood barrier; IADL = Instrumental activities of daily living; ICAT = individualised computerised anomia training; IFG = inferior frontal gyrus; MMSE = mini mental-state examination; MoCA = Montreal Cognitive Assessment; MRI = magnetic resonance imaging; NPI = Neuropsychiatric inventory; TPS = transcranial pulse stimulation.

## Data Availability

No new data were created or analyzed in this study. Data sharing is not applicable to this article.
